# Advances in Spectrometric Techniques in Food Analysis and Authentication

**DOI:** 10.3390/foods12030438

**Published:** 2023-01-17

**Authors:** Daniel Cozzolino

**Affiliations:** Centre for Nutrition and Food Sciences, Queensland Alliance for Agriculture and Food Innovation, St. Lucia Campus, The University of Queensland, Brisbane, QLD 4072, Australia; d.cozzolino@uq.edu.au

The demand from the food industry and consumers for analytical tools that can assure the quality (e.g., composition) and origin of foods (e.g., authenticity, fraud, provenance) in both the supply and value chains has increased over the past decades. Although there have been advances and improvements in analytical instrumentation and techniques that have excellent diagnostic capabilities, most of the existing routine methods of analysis are considered time-consuming and expensive. These issues have encouraged developments in the application of a wide range of spectrometric techniques, involving, among others, the utilization of vibrational spectroscopy combined with data analytics (e.g., chemometrics).

This Special Issue, “Advances in Spectrometric Techniques in Food Analysis and Authentication”, has compiled novel and recent applications of spectrometry-based techniques, including NIR, MIR, NMR, as well as other analytical techniques (e.g., ICP-MS and GC-MS) combined with chemometrics methods, to target issues associated with food analytics and authentication along the food supply and value chains (e.g., fraud, provenance, traceability).

In this Special Issue, Gajek and collaborators [1,2] have shown how the combination of chemometrics with ICP-MS data can be used to authenticate whisky samples. Chavez-Angel and collaborators have also described how vibrational spectroscopy (e.g., Raman and infrared) and thermal analysis can be combined to authenticate extra virgin olive oil [3]. The classification of coffee samples was also reported by combining both NIR and MIR spectroscopy with chemometric methods [4]. The utilization of spectral fingerprints was evaluated as a potential tool to screen the adulteration of traditional and Bourbon barrel-aged maple syrups [5]. The use of portable NIR instrumentation was also reported to discriminate and characterize individual goat muscles [6] and to differentiate meat species using a similarity index [7]. The use of a liquid–liquid microextraction method to enhance the flavor of seafood using GC-MS analysis [8], the analysis of saliva using MIR spectroscopy obtained from a sensory study [9], and the determination of the saponification value of fats and oils using 1H-NMR were also reported [10] in this Special Issue.

Overall, these applications have highlighted the importance of combining rapid analytical methods with chemometrics to improve our knowledge and understanding about foods.

## Data Availability

Not applicable.
